# Antithrombotic Therapy and Freedom From Bridging Stent Occlusion After Elective Branched Endovascular Repair: A Multicenter International Cohort Study

**DOI:** 10.1177/15266028241253133

**Published:** 2024-05-26

**Authors:** Vaiva Dabravolskaite, Lorenz Meuli, Ozan Yazar, Lee Bouwmann, Hozan Mufty, Geert Maleux, Pekka Aho, Harri Hakovirta, Maarit Venermo, Vladimir Makaloski

**Affiliations:** 1 Department of Vascular Surgery, Inselspital, University of Bern, Bern, Switzerland; 2 Department of Vascular Surgery, Turku University Hospital, Turku, Finland; 3 Satasairaala Hospital, Pori, Finland; 4 Department of Vascular Surgery, University Hospital Zurich, Zürich, Switzerland; 5 Department of Surgery, Zuyderland Medical Centre, Heerlen, The Netherlands; 6 Department of Vascular Surgery, Leuven University Hospital, Leuven, Belgium; 7 Department of Radiology, Leuven University Hospital, Leuven, Belgium; 8 Department of Vascular Surgery, Helsinki University Hospital, Helsinki, Finland

**Keywords:** antithrombotic therapy, branched endovascular aortic repair, bridging stent occlusion, low occlusion rate, higher occlusion rate for renal artery bridging stents

## Abstract

The risk of bridging stent occlusion after branched endovascular aortic repair (BEVAR) remains an issue. Currently, there is no clear recommendation on what kind of antithrombotic therapy should be installed after BEVAR. The aim of the study was to estimate the impact of postoperative antithrombotic therapy on bridging stent occlusion rate after elective BEVAR. An international multicenter retrospective analysis was performed in 4 European tertiary vascular units. All reno-visceral target vessels treated with bridging stents of patients undergoing elective BEVAR with the use of off-the-shelf or custom-made branched stent-grafts for pararenal aortic aneurysms (PAAs), type Ia endoleaks after previous EVAR, and thoracoabdominal aortic aneurysms (TAAA) between January 2014 and December 2022 were included. Primary outcome was freedom from bridging stent occlusion and its correlation with postoperative antithrombotic therapy. Secondary outcomes were overall survival and identifying target vessel and bridging stent characteristics, which may have a higher risk for bridging stent occlusion according to the PRINCE2SS recommendation. Follow-up information was obtained for all patients per 31st of December 2022. In total, 120 patients (90 male) with a median age of 72 (interquartile range [IQR]=67-77) years were included. Two hundred eighty-nine external and 127 internal branches were used for 416 target vessels. The median follow-up was 21 months (IQR=9-48) with a follow-up index of 1.0. During follow-up, 24 (5.8%) primary bridging stent occlusions (left renal artery [LRA]=10, right renal artery [RRA]=7, superior mesenteric artery [SMA]=3, truncus coeliacus [TC]=4) were found. The risk of renal bridging stent occlusion is significantly higher compared with visceral bridging stent, p=0.013. The occlusion rate was 7.8% for renal branches and 1.5% for visceral branches at 1 year and 10.6% and 3.7% at 5 years, respectively. The multivariable Cox proportional hazard model on bridging stent occlusion showed that there was no significant difference between the used antithrombotic strategies. No antithrombotic therapy was significantly associated with bridging stent occlusion, whereas no evidence for superiority of any other antithrombotic therapy was found. Overall, the bridging stents’ occlusion rate was low. We found a significantly higher occlusion rate in renal arteries compared with the visceral ones.

**Clinical Impact:**

Based on our study, no antithrombotic therapy is significantly associated with bridging stent occlusion, and no evidence of the superiority of other antithrombotic therapy exists. Nevertehless, due to the low number of bridging stent occlusions, this study can neither support nor reject the PRINCE2SS recommendations. Further studies with larger cohorts are needed to determine clear guideliness of the best antithrombotic treatment regimen after complex enfovascular aortic repair.

## Introduction

The success rate and durability of branched endovascular aortic repair (BEVAR) have significantly improved in the last decades. However, the risk of bridging stent occlusion remains an issue, with a prevalence of renal branch occlusion of up to 10% reported in the literature.^1,2^

Generally, the choice of bridging stent does not influence primary branch patency nor it is related to branch-related endoleak. Nevertheless, the self-expandable bridging stents for the renal arteries have lower rates of overall target vessel instability and reintervention.^3^ Using multiple stents and different stent types on the same target vessel could be associated with bridging stent fracture, thus leading to branch occlusion.^4^ Extensive branch length of more than 100 mm and tortuosity index >1.15 seems to increase the risk of branch instability, which may result in more diligent monitoring during follow-up.^5^

Currently, there is no clear recommendation on what kind of antithrombotic therapy should be installed after BEVAR. The recent European clinical practice guidelines on antithrombotic therapy for vascular disease do not consider any postoperative therapy for the bridging stents in visceral and renal arteries after complex endovascular treatment. A short course, ie, 1 month of dual antiplatelet therapy (DAPT), is recommended after post-revascularization for atherosclerotic renal or mesenteric disease.^6^ The PRINCE2SS International Expert-Based Delphi Consensus suggests a lifelong antithrombotic therapy, depending on target vessels’ anatomical characteristics, the use of multiple stents, and the stent’s length.^7^ However, the underlying evidence leading to this suggestion is low, and the risk of bleeding events should not be underestimated.^8^ In this multicenter international study, we aim to analyze the risk of bridging stent occlusion after elective BEVAR and the impact of postoperative antithrombotic therapy on the occlusion rate.

## Materials and Methods

### Study Design and Data Collection

An international multicenter retrospective analysis was performed from a prospectively collected database in 4 European vascular units. None of these vascular units or the authors were involved in the PRINCE2SS Delphi Consensus study. All reno-visceral target vessels treated with bridging stents of patients undergoing elective BEVAR between January 2014 and December 2022 were identified and included. Off-the-shelf or custom-made branched stent-grafts for pararenal aortic aneurysms (PAAs), type Ia endoleaks after previous EVAR, and thoracoabdominal aortic aneurysms (TAAAs) were used. Urgent and emergency BEVARs and patients treated solely with fenestrated stent-grafts were excluded. In case of a combination of fenestrations and branches, the target vessels treated with fenestrations were excluded from the analysis.

### Outcomes and Follow-up

The primary outcome was freedom from bridging stent occlusion and its association with the type and duration of postoperative antithrombotic therapy. Secondary outcomes were overall survival and identifying target vessel and bridging stent characteristics, which may have a higher risk for bridging stent occlusion according to the PRINCE2SS recommendation: vessel diameter <6 mm, tortuosity >60° within 30 mm from the origin of the target vessel, use of multiple stents, and total stent’s length used >50 mm. Total stent length comprised the stent overlap in the branch, free part in the sac, and inside the target vessel. Follow-up information on bridging stent-graft patency, reinterventions, antithrombotic therapy, and survival was obtained for all patients and for each target vessel via hospital records and through a cross-sectional telephone survey with patients/relatives or general practitioners until the 31st of December 2022. All patients were followed after 3, 6, and 12 months and yearly there after index operation with a computed tomography angiography (CTA). In case of clinical presentation correlation with a bridging stent occlusion, urgent CTA and open or endovascular treatment followed. If a bridging stent occlusion was detected in a regular follow-up and an asymptomatic patient, its potential treatment was left to discretion of the treated surgeon. Completeness of follow-up was quantified using the follow-up index (FUI) for each patient.^9^

### Definitions

Antithrombotic therapy was categorized into aspirin, clopidogrel, DAPT, oral anticoagulation (OAC) + mono antiplatelet therapy, OAC alone, or no antithrombotic therapy. Oral anticoagulation included direct factor X inhibitors (DOAC) or vitamin K antagonists (VKAs). Antithrombotic therapy was reported individually by time intervals where different treatment regimen was prescribed. Arterial hypertension was defined as a baseline blood pressure >140/90 mmHg and/or the intake of one or more antihypertensive drugs. Hyperlipidemia was defined as abnormal cholesterol or triglycerides blood level and/or the intake of lipid-lowering drugs. Chronic kidney disease was defined as a reduction of kidney function (glomerular filtration rate [GFR] <60 mL/min) according to the KDIGO 2017 Clinical Practice Guideline.^10^ Increased creatinine level at discharge more than 20% compared with the values at admission as well the need for transient or permanent dialysis was defined as postoperative renal function deterioration. Peripheral artery disease was defined as a history of lower limbs surgical or endovascular revascularization and/or clinical symptoms such as claudication, rest pain or foot ulcers, and/or abnormal ankle/brachial index (ABI), and duplex scan findings.

### Procedural Details

The elective repair was indicated if an aneurysm diameter was ≥55 mm for the PAA and ≥60 mm for all types of TAAA, including postdissection aneurysms. In all 4 vascular units, either off-the-shelf (T-branch, Cook Medical, Bloomingdale, Illinois or E-nside, Jotec/Artivion, Hechingen, Germany) or custom-made branched stent-grafts (CMD, Cook and E-Xtra design, Jotec/Artivion) were used. Material used depended on institutional availability and policy and was left to the discretion of the treating surgeon. Each bridging stent’s type, length, and diameter were retrieved from perioperative protocols. Furthermore, the total length (ie, if more than 1 stent was used) was measured on postoperative computed tomography (CT) scans. Perioperative cerebrospinal fluid drainage (CSFD) depended on the covered aortic length, previous aortic surgery, and whether the procedure was staged.

### Statistics

Baseline characteristics of patients were presented using tables and summary statistics. Continuous variables were summarized with median and quartiles (Q1, Q3) if skewed and with mean and standard deviation (SD) if normally distributed. Characteristics of bridging stents are presented separately and stratified by target vessel. Time to bridging stent occlusion was analyzed using a cumulative incidence function with death as a competing risk and compared between the type of target vessel (renal vs visceral) using Gray’s test. Furthermore, a Cox proportional hazard model with the antithrombotic therapy (factor) as a time-varying covariate and mortality as a competing risk was conducted. All time intervals of patients on a specific antithrombotic therapy were recorded and included. The model was adjusted for the total stent length, the target vessel diameter ≥6 mm (binary), and vessel tortuosity >60° (binary). The target vessel and bridging stent characteristics were chosen according to the PRINCE2SS recommendation. The proportional hazard assumption was tested and verified using scaled Schoenfeld residuals. The time-to-event analyses included each bridging stent separately. Thus, these analyses allowed for non-independent observations as every patient was included up to 4 times (once for each bridging stent). An alpha level of 5% was predefined. This retrospective observational study is reported following the Strengthening the Reporting of Observational Studies in Epidemiology (STROBE) Statement.^11^

## Results

### Patient Management

**Figure 1.**
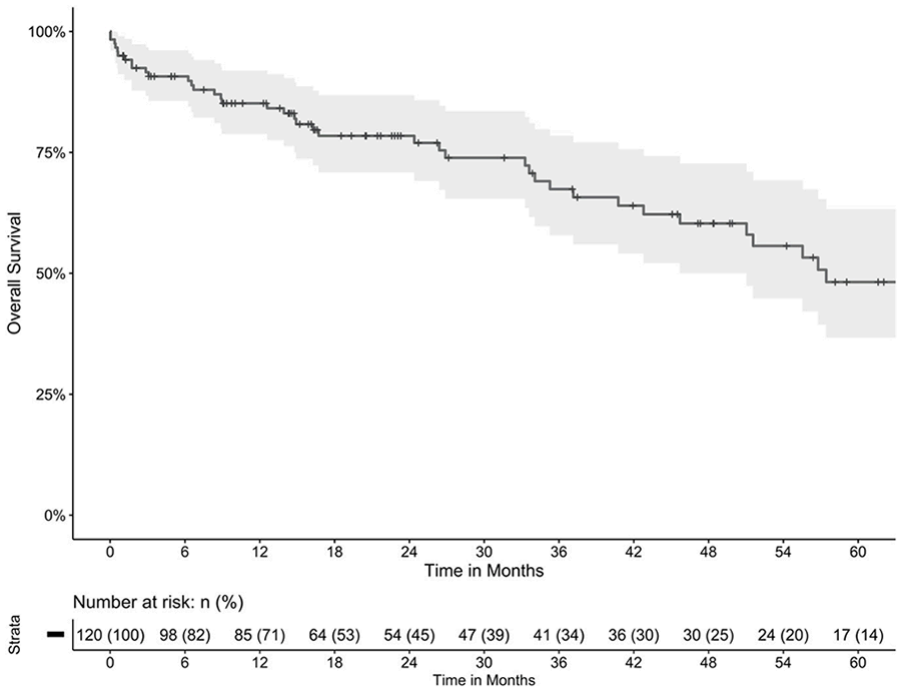
Estimated overall survival. Estimated overall survival of 85% (95% CI=79%-92%) at 1 year and 48% (37%-63%) at 5 years.

In total, 120 patients (75% male) with a median age of 72 (67-77) years underwent elective BEVAR. Patients’ baseline characteristics are presented in Table 1. The preoperative antithrombotic therapy included aspirin in 66 patients (55.0%), clopidogrel in 16 patients (13.3%), and 26 patients (21.7%) on OAC, respectively. All 120 BEVARs were performed in general anesthesia. Two hundred eighty-nine outer branches were used in 87 (72.5%) patients outer and 127 inner branches in 33 (27.5%) patients. Inner branch designs were only used in 3-fold or 4-fold BEVAR designs. Cerebrospinal fluid (CSF) drainage was used in 51 patients (42.5%). Bridging stents were completed via upper extremity access in 84 patients (70.0%), via femoral access in 30 patients (25.0%), and in 6 patients (5.0%); both routes were used. The median operating time was 290 minutes (233-365) with a median blood loss of 400 mL (300-700). Postoperatively, 44 patients (36.7%) had aspirin only, 8 patients (6.7%) had clopidogrel only, and 57 patients (47.5%) were prescribed DAPT. Thirty-one patients (25.8%) were prescribed OAC. Procedural details, postoperative management, and hospital mortality are presented in Table 2. Follow-up information was complete, indicated by an FUI of 1.0. The median hospital stay was 6 days (5-9), and the all-cause in-hospital mortality rate was 3.4% (4 of 120). During follow-up, 42 patients died, resulting in an estimated overall survival of 85% (95% confidence interval [CI]=79%-92%) at 1 year and 48% (37%-63%) at 5 years (Figure 1).

**Table 1.** Baseline Characteristics.

	Total (N=120)
**Male sex, *n (%)***	90 (75.0)
**Age, *years***	72 (67, 77)
**Preoperative antiaggregant**	
ASA, *n (%)*	66 (55.0)
Clopidogrel, *n (%)*	16 (13.3)
Ticlopidine, *n (%)*	1 (0.8)
Ticagrelor, *n (%)*	1 (0.8)
None, *n (%)*	36 (30.0)
**Preoperative OAC**	
DOAC, *n (%)*	15 (12.5)
VKA, *n (%)*	11 (9.2)
**BMI, *kg/m*** ^2^	25 (22, 29)
**Ever smoking, *n (%)***	81 (76.4)
Missing, *n*	14
**Active smoking, *n (%)***	24 (22.4)
Missing, n	13
**Diabetes mellitus, *n (%)***	23 (19.5)
Missing, n	2
**Chronic kidney disease, *n (%)***	38 (31.7)
**COPD, *n (%)***	29 (24.2)
**Coronary artery disease, *n (%)***	45 (37.5)
**Atrial fibrillation, *n (%)***	25 (20.8)
**Peripheral artery disease, *n (%)***	16 (13.3)
**Malignancy, *n (%)***	30 (25.0)
**Previous thoracic aneurysm repair**	
No previous repair, *n (%)*	17 (14.2)
Endovascular repair, *n (%)*	26 (21.7)
Open repair, *n (%)*	16 (13.3)
Hybrid repair, *n (%)*	61 (50.8)
**Mobility without assistance, *n (%)***	116 (96.7)

ASA: acetylsalicylic acid; OAC: oral anticoagulation; DOAC: direct oral anticoagulant; VKA: vitamin K antagonist; BMI: body mass index; COPD: chronic obstructive pulmonary disease.

Data were complete if not stated explicitly. Continuous variables are presented by median and (quartiles 1, 3). Counts are presented with numbers and (percentages). Factor variables were compared by the chi-square test and continuous variables by the Kruskal-Wallis rank test, respectively.

Chronic kidney disease and peripheral arterial disease as defined in section “Materials and Methods.”

**Table 2.** Procedural Details and Outcomes.

	Total (N=120)
**Branch design**	
Outer, n (%)	87 (72.5)
Inner, *n (%)*	33 (27.5)
**Endograft type**	
Artivion E-nside	9 (7.5)
CMD Artivion	41 (34.2)
CMD Cook	29 (24.2)
Cook t-Branch	41 (34.2)
**CSF drainage, *n (%)***	51 (42.5)
**Access for bridging stents**	
Upper extremity, *n (%)*	84 (70.0)
Femoral only, *n (%)*	30 (25.0)
Both, *n (%)*	6 (5.0)
**OP duration, *minutes***	290 (233, 365)
**Blood loss, *mL***	400 (300, 700)
**Postoperative antiaggregant**	
ASA only, *n (%)*	44 (36.7)
Clopidogrel only, *n (%)*	8 (6.7)
DAPT, *n (%)*	57 (47.5)
None, *n (%)*	11 (9.2)
**Postoperative OAC, *n (%)***	31 (25.8)
**Length of stay, *days***	6 (5, 9)
**Hospital mortality, *n (%)***	4 (3.4)
Missing, *n*	1
**Follow-up, *months***	21 (9, 48)
**Follow-up Index**	1

CMD: custom-made design; CSF: cerebrospinal fluid; OP: operation; ASA: acetylsalicylic acid; DAPT: dual antiplatelet therapy; OAC: oral anticoagulation.

Data were complete if not stated explicitly. Continuous variables are presented by median and (quartiles 1, 3). Counts are presented with numbers and percentages. Factor variables were compared by chi-square test and continuous variables by the Kruskal-Wallis rank test, respectively.

### Target Vessels and Bridging Stents

A total of 416 target vessels were successfully treated with bridging stents, of which 97 were left renal arteries (LRA), 101 right renal arteries (RRA), 116 superior mesenteric arteries (SMA), and 102 celiac trunks. Vessel diameters were significantly smaller in renal arteries compared to visceral arteries (p<0.001), and vessel tortuosity was significantly more often seen in renal than visceral arteries (p<0.001). Balloon-expandable stents (83.4%) were preferred over self-expandable stents (16.6%). This preference was similar for all target vessels (p=0.988). According to the target vessel diameters, stent diameters were significantly smaller in renal arteries than visceral ones (p<0.001). Primary relining was performed in 25% of all bridging stents, and this percentage did not differ significantly between the target vessels. More details of all bridging stents are presented in Table 3.

**Table 3.** Bridging Stent.

	LRA (N=97)	RRA (N=101)	SMA (N=116)	Celiac trunk (N=102)	p-value
**Vessel diameter, mm**	5.6 (5.0, 6.5)	5.5 (5.0, 6.0)	8.0 (7.0, 8.9)	7.8 (7.0, 8.8)	<0.001
<6mm, *n (%)*	55 (56.7)	61 (60.4%)	6 (5.2%)	4 (3.9%)	<0.001
≥6mm, *n (%)*	42 (43.3)	40 (39.6%)	110 (94.8%)	98 (96.1%)	
**Vessel tortuosity ≥60°, *n (%)***	31 (32.0)	43 (42.6%)	5 (4.3%)	16 (15.7%)	<0.001
**Stent type**					0.988
Self-expandable, n (%)	17 (17.5)	17 (16.8%)	19 (16.4%)	16 (15.7%)	
Balloon-expandable, n (%)	80 (82.5)	84 (83.2%)	97 (83.6%)	86 (84.3%)	
**Stent brand**					0.977
Artivion E-ventus BX, *n (%)*	34 (35.1)	38 (37.6%)	38 (32.8%)	39 (38.2%)	
BD Fluency, *n (%)*	17 (17.5)	16 (15.8%)	17 (14.7%)	15 (14.7%)	
BD LifeStream, *n (%)*	0 (0.0)	0 (0.0%)	1 (0.9%)	0 (0.0%)	
Bentley BeBraft, *n (%)*	2 (2.1)	2 (2.0%)	1 (0.9%)	1 (1.0%)	
Bentley BeBraft PLUS, *n (%)*	5 (5.2)	5 (5.0%)	6 (5.2%)	3 (2.9%)	
Getinge Advanta V12, *n (%)*	10 (10.3)	5 (5.0%)	8 (6.9%)	6 (5.9%)	
Gore Viabahn VBX, *n (%)*	28 (28.9)	32 (31.7%)	40 (34.5%)	34 (33.3%)	
Combination, *n (%)*	1 (1.0)	3 (3.0%)	5 (4.3%)	4 (3.9%)	
**Stent diameter**					<0.001
≤5 mm, *n (%)*	10 (10.6)	10 (9.9)	0 (0.0)	1 (1.0)	
6 mm, *n (%)*	45 (47.9)	58 (59.2)	4 (3.5)	7 (7.0)	
7 mm, *n (%)*	29 (30.9)	23 (23.5)	23 (20.4)	21 (21.0)	
8 mm, *n (%)*	6 (6.4)	7 (7.1)	37 (32.7)	35 (35.0)	
9 mm, *n (%)*	2 (2.1)	0 (0.0)	20 (17.7)	11 (11.0)	
≥10 mm, *n (%)*	2 (2.1)	0 (0.0)	29 (25.0)	25 (24.5)	
Missing, *n*	3	3	3	2	
**Stent length**					0.001
22-29 mm, *n (%)*	1 (1.0)	2 (2.0)	0 (0)	1 (1.0)	
37-39 mm, *n (%)*	3 (3.2)	14 (14.2)	2 (1.7)	16 (16.0)	
57-59 mm, *n (%)*	65 (69.1)	62 (63.2)	75 (65.7)	64 (64.0)	
≥59 mm, *n (%)*	25 (26.6)	20 (20.4)	37 (32.4)	19 (19.0)	
Missing, *n*	3	3	2	2	
**Total number of stents**					0.531
n=1, *n (%)*	65 (67.0)	76 (75.2)	90 (77.6)	81 (79.4)	
n=2, *n (%)*	28 (28.9)	21 (20.8)	19 (16.4)	16 (15.7)	
n≥3, *n (%)*	4 (4.1)	4 (4.0)	7 (6.0)	5 (4.9)	
**Total stent length, *mm***	57 (54, 75)	55 (54, 70)	59 (56, 70)	56 (53, 62)	0.017
**Primary stent relining, *n (%)***	32 (33.0)	25 (24.8)	26 (22.4)	21 (20.6)	0.189

LRA: left renal artery; RRA: right renal artery; SMA: superior mesenteric artery.

Data were complete if not stated explicitly. Continuous variables are presented by median and (quartiles 1, 3). Counts are presented with numbers and (percentages). Factor variables were compared by chi-square test, and continuous variables by the Kruskal-Wallis rank test, respectively.

### Bridging Stent Occlusion

**Figure 2.**
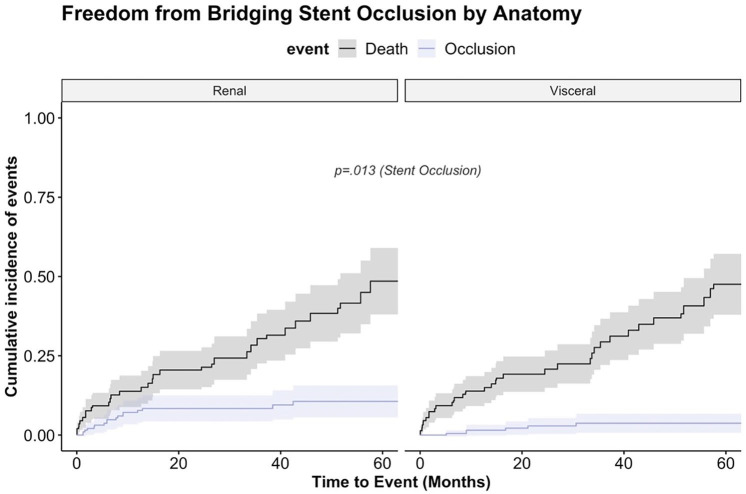
Bridging stent occlusion (competing risk analysis). Cumulative incidence function of bridging stent occlusion and mortality as a competing risk stratified by reno-visceral target vessel. The occlusion rate was significantly higher in renal bridging stent-grafts (p=0.013). Survival was for both groups similar: at 12 months, 86%, and at 60 months, 52% (p=0.777) (cave: non-independent observations).

The median follow-up was 21 months (9.1, 47.6). Follow-up information was complete, FUI 1.0. During the follow-up, we found 8.7% target vessel instability, 12 (2.9%) patent bridging stents required reintervention due to stent fracture, kinking or in-stent stenosis with secondary relining and 24 (5.8%) bridging stents occluded (LRA=10, RRA=7, SMA=, celiac trunk=4). The risk of renal bridging stent occlusion was significantly higher compared with visceral bridging stent, p=0.013 (see Figure 2). The occlusion rates were at 1 year 7.8% (95% CI=3.5%-11.7%) for renal branches and 1.5% (0.0%-3.3%) for visceral branches, at 5 years 10.6% (5.6%-15.7%) for renal branches and 3.7% (0.7%-6.8%) for visceral branches. Sixteen of the 24 occluded branches were outer (5.5%, 16/289) and 8 inner branches (6.2%, 8/127). The multivariable Cox proportional hazard model on bridging stent occlusion showed that there was no significant difference between the used antithrombotic strategies, except for no antithrombotic therapy being associated with an almost 11-fold increased risk for stent occlusion (hazard ratio [HR]=10.7, 95% CI=1.12-102, p=0.039) (see Table 4). The model was adjusted for total stent length, use of multiple stents, target vessel diameter, and target vessel tortuosity. Total stent length, HR=1.03 (1.01-1.06; p=0.015), was significantly associated with stent occlusion. The binary variables use of multiple stents, target vessel diameter, and target vessel tortuosity were not associated with stent occlusion. The median time to graft occlusion was 260 (144-422) days. Many occlusions occurred during aspirin monotherapy after an initial uneventful treatment with DAPT. However, this observation did not lead to any statistically significant result. Characteristics of all occluded bridging stent-grafts and their target vessels are presented in the Supplementary Table S1. Twenty-one of the 24 occluded bridging stents were part of a 4-fold implanted branched stent-graft; 1 was a 3-fold branches stent-graft, and the other 2 had a single branch for 1 renal artery. Six of the 24 occluded bridging stents were self-expanding, and 18 were balloon-expandable bridging stents. Concerning the PRINCE2SS recommendation, of the 24 occlusions, a target vessel diameter <6 mm was found in 10, tortuosity >60° in 7, and a total stent length used of >50 mm in 21 patients.

**Table 4.** Bridging Stent Occlusion (Multivariable Analysis).

	HR	95% CI	p-value
**Antithrombotic management**			
ASA monotherapy	Reference		
Clopidogrel monotherapy	2.29	0.70 to 7.51	0.171
DAPT	1.64	0.51 to 5.24	0.402
OAC + monotherapy	2.25	0.73 to 6.96	0.159
OAC only	0.00	0.00 to 0.00	NA
None	10.7	1.12 to 102	0.039
**Total stent length, mm**	1.03	1.01 to 1.06	0.015
**Vessel diameter ≥6 mm**	0.53	0.20 to 1.43	0.211
**Tortuosity ≥60°**	1.29	0.44 to 3.75	0.640

HR: hazard ratio; 95% CI: 95% confidence interval; ASA: acetylsalicylic acid; DAPT: dual antiplatelet therapy; OAC: oral anticoagulation.

Time-to-event analysis using a Cox proportional hazard model with antithrombotic management as a time-varying covariate and mortality as a competing risk. ASA monotherapy, vessel diameter <6 mm and non-tortuosity served as reference groups. No stent occlusion was observed for the time interval with OAC monotherapy; thus, there is a statistical separation, and the estimates are not meaningful.

## Discussion

Complex endovascular aortic repair with branched stent-grafts has been a first-line treatment option for different thoracoabdominal aortic pathologies for more than 10 years. However, there is no high evidence data to support 1 antithrombotic treatment regime over other after BEVAR. Driven by this fact, an international expert-based Delphi consensus group recently released a summary of recommendations based on their personal experience.^7^ In this retrospective multicenter international analysis initiated by the current PRINCE2SS recommendation, we report the freedom of bridging stent occlusion after elective BEVAR correlating with the postoperative antithrombotic regimens. The overall bridging stent occlusion rate was very low. Similar to previous reports, we found a significantly higher rate of occluded renal bridging vs visceral bridging stents.^1,12,13^

Most of the occlusions occurred during the first year of follow-up, independently of the antithrombotic regimens, with some being on DAPT or OAC at the moment of occlusion. Due to the small number of occlusions in our series, we could not show any correlation with the postoperative antithrombotic regimens. Based on our data, we cannot justify the occlusion of 1 bridging stent in a patient with 4 branches due to an inappropriate antithrombotic regimen.

A recent meta-analysis of current retrospective studies suggests lower overall target vessel instability and reintervention rates favor the self-expanding bridging stents.^3^ We found no difference in our series comparing the types of occluded bridging stents, 5.2% (18/346) balloon-expandable vs 8.6% (6/70) self-expandable stents. The PRINCE2SS recommendations do not differ between antithrombotic therapy for self- or balloon-expandable stents but suggest postoperative lifelong DAPT, in case branches are mated with small (<6 mm) or highly tortuous target vessels or multiple/long stents are employed. The analysis of the 24 occluded bridging stents showed different target vessel/bridging stents characteristics, varying from 4.7 mm diameter of a renal artery up to 11 mm diameter of an occluded, with the use of multiple stents in only 11 from 24 occluded bridging stents and total stents length used between 34 and 115 mm. The majority of the occluded vessels and their mating bridging stents in our series were not at risk for occlusions despite PRINCE2SS recommendations (Supplementary Table S1); moreover, most of the patients were on extensive antithrombotic treatment at the time of the occlusion (Table 4). A more profound analysis of all mechanical, especially hemodynamic/pathophysiological changes in the target vessel, might reveal more understanding of the unknown area of bridging stent occlusion in BEVAR. A very recent analysis of some mechanical components with 3D geometric models constructed from CTA during both inspiratory and expiratory breath-holds, pre- and post-operatively, showed that reduction in respiratory-induced deformation of branch take-off angle from pre- to post-BEVAR should reduce the risk of device disengagement and endoleak.^14^ The unchanged respiratory-induced end-stent bending should maintain the native vessel dynamics distal to the bridging stents, thus potentially resulting in lower fatigue risk and stent occlusion. Opposite to the PRINCE2SS recommendations, this study concludes that using longer bridging stent paths in BEVAR may enable smoother paths subject to less dynamic bending and allow for a lower bridging stent occlusion rate. Piazza et al^5^ recommended that the total branch length covered by self-expandable bridging stents, including branch overlap and target vessel landing zone in BEVAR for TAAA, should be between 60 and 100 mm. Shorter and longer total branch lengths were associated with branch instability. In our series, 6 self-expandable bridging stents occluded, all having a total length between 60 and 100 mm and only 1 with severe tortuosity; therefore, we could not confirm this observation. Hauck et al^2^ reported a higher structural failure rate of single-layer balloon-expandable bridging stents in 54 patients undergoing BEVAR between 2012 and 2020. They detected 12% (23/185) of bridging stents with structural failure (leaks, stent fractures, and complete stent disruption) in 12 patients, with a higher incidence between 2014 and 2019. During this period, most covered balloon-expandable stents used as bridging stents had a single-layer ePTFE membrane and cobalt chromium or stainless-steel bare stent core. Similarly, all our occluded bridging balloon-expandable stents had a single-layer ePTFE membrane with a bare stent core and happened between 2015 and 2020. An undetected and untreated structural failure of a bridging stent might lead to an occlusion. After introducing the double core stent with double-layered ePTFE membrane and the new generation of single-layered ePTFE stents and their broader use in BEVAR, the rate of bridging stents occlusion significantly decreased.^15,16^ Between 2020 and 2022, the number of BEVARs in all 4 centers increased and no further bridging stent occlusion was observed. This might add an additional explanation to our low bridging stent occlusion rate in total.

Several studies reported similar rates of in-hospital mortality during follow-up. Oderich et al^17^ had in a cohort of 185 patients a 30-day mortality rate of 4.3%. After a mean follow-up of 22 ± 20 months, the authors found that the patient’s survival rate was 57.5% at 5 years. There was 1 late aneurysm-related death due to an untreated aortic segment rupture and no information about deaths during follow-up due to antithrombotic-induced bleeding. A bigger series with 468 patients over a longer period (2004-2016) reported a low 30-day mortality rate of 4.9%, an estimated survival rate of 59.6% after 5 years, and a median follow-up of 29 months.^18^ We found a similar low in-hospital mortality rate of 3%. Still, after a median follow-up of 21 months, the estimated 5-year survival rate in our series of 46% was significantly lower compared with other studies. Our cohort has the same median age of 72 years as the other 2 studies. Still, of the 42 deaths during follow-up, 31 patients (74%) had previous open and/or endovascular TAAA repair, meaning that this population was already severely diseased prior BEVAR. According to van Calster et al,^18^ previous type I to III TAAA is a significant independent risk factor for late mortality.

### Limitations and Strengths

The retrospective nature of this study might be a potential limitation. Furthermore, the Cox proportional hazard model was adjusted for stent diameter as a main cofounder, but due to the limited number of patients and low number of events with the potential of a statistical separation, no further potential confounders were included—missing cause of death, including possible antithrombotic-induced bleeding, etc. Patients’ compliance with their medicaments remains an open question for any study analyzing the influence of antithrombotic therapy on stent patency, and therefore, it is difficult to prove it retrospectively. Different antithrombotic regimens may influence the stent’s patency and increase the risk of antithrombotic-induced bleeding. No sufficient data were available to reflect the bleeding-related complications from all patients during follow-up. Therefore, we were not able to present this data and balance the discussion. Although no antithrombotic therapy was associated with a higher risk of graft occlusion, for the “OAC only” group, a separation problem occurred. In this very small group, no events occurred, and therefore, no exact estimates are possible. During the study period, there was a shift from self-expandable to balloon-expandable stents in some centers. As a result, there is a difference in follow-up time, as self-expandable stents had a longer follow-up.

Despite the fact that 4 centers with different treatment strategies and materials used were included, we found no differences in outcomes. The status of bridging stent’s patency was reported based on regular follow-up CT scans and additional CT scans performed for symptomatic patients. As a result, we truly believe that we identified all potential issues related to bridging stents. All included centers follow-up patients after BEVAR very closely, which resulted in a complete FUI of 1.0 and detailed outcome for every patient, including exact timing of branch occlusion, antithrombotic therapy regime at the moment of occlusion, and patency.

## Conclusions

The incidence of bridging stent occlusion is very low. The bridging stents for the renal arteries have a significantly higher occlusion rate than the visceral ones. According to our study, no antithrombotic therapy is significantly associated with bridging stent occlusion, and no evidence of the superiority of other antithrombotic therapy exists. However, due to the low number of bridging stent occlusions, this study can neither support nor reject the PRINCE2SS recommendations. Increased bridging stent occlusion was seen in patients who had no antithrombotic medication. Further studies with larger cohorts are needed to determine the best antithrombotic treatment regimen after BEVAR.
